# The Role of Socioeconomic Inequalities In Transitioning to Neurocognitive Disorders in English Population

**DOI:** 10.1093/geroni/igab046.2670

**Published:** 2021-12-17

**Authors:** Aswathikutty Gireesh, Amanda Sacker, Anne McMunn, Dorina Cadar

**Affiliations:** 1 University College London, London, England, United Kingdom; 2 University College London (UCL), London, England, United Kingdom

## Abstract

The association between socioeconomic position (SEP) and dementia is well studied. However, scant attention has been given to the relationship with mild cognitive impairment (MCI), often considered a transient state between normal cognition and dementia. The purpose of this study was to determine the role of various SEP markers such as education and wealth on transitioning to MCI and dementia over a four-year period using data from the English Longitudinal Study of Ageing, a national representative sample of the English population aged 50+. We ascertained MCI and dementia over four years, using a validated algorithm based on physician diagnosis and lower cognitive performance (1 standard deviation below the mean) on multiple standardised tests adjusted for age and education. A Multistate Markov survival model was utilised to investigate whether different SEP markers increased the risk of specific transitions between normal cognitive performance and MCI or dementia, with the latter being considered an absorbing state. During the study period, a quarter of participants progressed to MCI from the normal state. Being in the lowest quintile of wealth was associated with a lower probability of transitioning back to a normal cognitive state from MCI, compared with those in the highest quintile. Greater wealth was weakly associated with a lower risk of transitioning from normal cognitive state to MCI and from MCI to dementia. The overall results imply that socioeconomic advantage might be protective against rapid progression from mild to more severe neurocognitive disorders such as dementia in later life.

